# Chemical Compositions before and after Lactic Acid Fermentation of Isoflavone-Enriched Soybean Leaves and Their Anti-Obesity and Gut Microbiota Distribution Effects

**DOI:** 10.3390/nu16111693

**Published:** 2024-05-29

**Authors:** Hee-Yul Lee, Du-Yong Cho, Jong-Bin Jeong, Ji-Ho Lee, Ga-Young Lee, Mu-Yeun Jang, Jin-Hwan Lee, Kye-Man Cho

**Affiliations:** 1 Department of Green Bio Science and Agri-Food Bio Convergence Institute, Gyeongsang National University, Jinju 52727, Republic of Korea; 2 Department of Life Resource Industry, Dong-A University, 37, Nakdong-Daero 550 Beon-gil, Saha-gu, Busan 49315, Republic of Korea

**Keywords:** isoflavone-enriched soybean leaves, lactic acid fermentation, isoflavones, free amino acids, anti-obesity, gut microbiota

## Abstract

In this study, we prepared fermented products of isoflavone-enriched soybean leaves (IESLs) and analyzed their nutrients, isoflavones, anti-obesity efficacy, and effects on gut microbiota. Fermented IESLs (FIESLs) were found to be rich in nutrients, especially lauric acid, oleic acid, and linoleic acid. In addition, the concentrations of most essential free amino acids were increased compared to those of IESLs. The contents of bioactive compounds, such as total phenolic, total flavonoid, daidzein, and genistein, significantly increased as well. In addition, FIESLs administration in a high-fat diet (HFD) animal model improved the final body weight, epididymal fat, total lipid, triglyceride, total cholesterol, blood glucose, and leptin levels, as well as reverting microbiota dysbiosis. In conclusion, these findings indicate that FIESLs have the potential to inhibit obesity caused by HFDs and serve as a modulator of gut microbiota, offering the prevention of diet-induced gut dysbiosis and metabolite diseases associated with obesity.

## 1. Introduction

Soybean leaves (SLs), a by-product of soybeans, are typically discarded, except in Korea. Commonly, residents of the Republic of Korea reserve SLs to be pickled. However, interest in their potential benefits has grown since it was reported that a greater variety of flavonoids is found in leaves than in seeds [1]. SLs are reported to include flavonols, and flavones include kaempferol glycosides [1]. Furthermore, several effects, such as anti-obesity [2], anti-inflammatory, and antioxidant effects, have been reported [3]. Several years ago, new SLs were produced using ethylene [4]. This product has more than five times the content of isoflavones in the form of glycosides compared with existing SLs. Recently, due to its value as a functional ingredient, efforts have focused on strengthening the aglycone form of isoflavones through fermentation using lactic acid bacteria (LAB) [5].

The World Health Organization (WHO) defines obesity as the excessive accumulation of fat in the body [6]. In modern society, the occurrence of obesity is rapidly increasing; according to the WHO, over 100 million people will be obese by 2030. The speed of this increase is dramatically faster than our prediction [7]. High-energy food intake and a lack of physical activity appear to be the main drivers of this phenomenon. Obesity causes cardiovascular disease, hyperlipidemia, hypertension, nonalcoholic fatty liver, rising insulin resistance, and type 2 diabetes [6]. Therefore, obesity prevention is essential; accordingly, interest in natural products or foods that control blood glucose and inhibit cell oxidative stress is increasing [8].

Gut microbiota can be considered as a host microbial organ that regulates host nutrient absorption, energy metabolism, and intestinal barrier function [2,6]. The dietary composition of a host affects the gut microbiota composition. Plant-based diets, particularly those rich in polyphenols, fibers, and carbohydrates, can modify the balance of gut microbiota in a host [9,10]. Recently, it has been reported that mulberry leaves have anti-obesity properties associated with the alteration of gut microbiota [8]. In addition, dietary plants such as fruits, vegetables, spices, legumes, grains, and tea have been reported to display anti-obesity abilities by increasing the diversity of gut microbiota and regulating either anti-obesity or obesogenic gut microbiota [9].

In this study, isoflavone-enriched soybean leaves (IESLs) were fermented with lactic acid bacteria, and its components before and after fermentation were compared. In addition, we evaluated the anti-obesity efficacy and blood sugar reduction in unfermented IESLs (UFIESLs) and fermented IESLs (FIESLs) on high-fat diet (HFD) models in C57BL/6J male mice. Additionally, we characterized their effects on gut microbiota. Therefore, we attempted to confirm the value of functional food test substances by verifying the effect of specific diets (UFIESLs and FIESLs) on obesity.

## 2. Materials and Methods

### 2.1. Plants, Bacteria, Chemicals, and Instruments

The IESLs used in this study were grown as previously reported [4,5] and were supplied in a dried state from JCN Farm Co. (Jinju, Gyeongsangnam-do, Republic of Korea). The LAB used in this study were *Lactiplantibacillus plantarum* P1201 and *Levilactobacillus brevis* BMK184 [5] strains, which were isolated from fermented foods such as *kimchi* and confirmed to have potential probiotic activity. The LAB cultures were liquid and solid cultures, using Lactobacilli MRS broth/agar, respectively (MRSB/MRSA, Difco, Becton Dicknson Co., Sparks, MD, USA). The isoflavone derivatives used were daidzein, glycitein, genistein, daidzin, glycitin, and genistin. Folin–Ciocalteu reagent, gallic acid, diethylene glycol, rutin, α-glucosidase, pancreatic lipase, *ρ*-nitrophenyl-α-D-glucopyranoside (*ρ*-NPG), and *ρ*-nitrophenyl-butyrate (*ρ*-NPB) were purchased from Sigma-Aldrich (St. Louis, MO, USA), as were all other chemicals.

The total phenolic contents (TPCs), total flavonoid contents (TFCs), and enzyme inhibition activities were determined using a Shimadzu UV–visible scanning spectrophotometer (UV-1800 240V, Shimadzu Co., Kyoto, Japan). A gas chromatograph (GC, Agilent 7890A system, Agilent Technologies Inc., Wilmington, DE, USA), automatic amino acid analyzer (L-8900, Hitachi High-Technologies Inc., Tokyo, Japan), and high-performance liquid chromatograph (HPLC, Agilent 1200 series, Agilent Co., Forest Hill, VIC, Australia) were used for the analysis of fatty acids, free amino acids, and isoflavones, respectively.

### 2.2. Fermentation of IESLs

A total of 500 g of distilled water was added to 500 g of IESLs, with 2.5% sugar, sterilized at 121 °C for 15 min, and cooled to 25 °C. The mixed strains *L. plantarum* P1201 and *L. brevis* BMK184 (1:2, *v*/*v*) were then inoculated and fermented at 30 °C for 72 h.

### 2.3. Physicochemical Properties

The pH was measured by adding 10 mL of distilled water to 1 g of samples, stirring, and using a pH measuring device (MP 200 pH meter, Mettler Toledo GmbH, Schwerzenbach, Switzerland). The acidity was measured by adding 50 mL of distilled water to 1 mL of the sample, shaking it, and neutralizing it with 0.1 N NaOH until the pH reached 8.2 ± 0.1; the amount consumed at this time was converted to lactic acid (%) using the following Formula (1):

Acidity (%, lactic acid) = [(N × V × Eq. wt)/(weight of samples × 1000)] × 100(1)

where N—normality of NaOH; V—volume of NaOH; Eq. wt—equivalent weight of lactic acid (CH_3_-CHOH-COOH, MW = 90); 1000 = factors transforming mL to L.

The reducing sugar and soluble protein content were measured by slightly modified DNS and Biuret methods, respectively [11]. The quantification of viable cells was conducted by inoculating a diluted suspension onto an MRS agar plate. After 48 h of incubation at 37 °C, we quantified the colony counts.

### 2.4. Analysis of Digestive Enzyme Inhibitory Activities

The digestive enzyme inhibitory activities of samples (UFIESLs and FIESLs) were measured using the method presented by Lee et al. [5], with slight modifications. A volume of 40 mL of 50% ethanol (EtOH) was used to extract the sample (2.0 g) over 24 h at 25 °C in a shaking incubator. The supernatant was centrifuged at 6000 rpm for 15 min and filtered using a membrane filter (0.45 μm, Whatman, Maidstone, UK). The extracts were concentrated in a rotary evaporator (N-1300, SHANGHAI EYELA Co., Ltd., Shanghai, China) at 60 °C and lyophilized. For α-glucosidase and pancreatic lipase inhibitory activities, lyophilized samples were prepared at concentrations of 0.25, 0.5, 0.75, and 1 mg/g with 50% EtOH. A measure of 3 mL of each sample was placed in a test tube with 70 μL of 1.0 U/mL enzyme solutions (α-glucosidase and pancreatic lipase). Next, 50 μL of 200 mM sodium phosphate buffer (pH 6.8) and 100 μL of 10 mM enzyme substrates (*p*-NPG and *p*-NPB in a buffer) were added to the mixture and incubated at 37 °C for 10 min. Finally, the reaction was stopped by 750 μL of 100 mM Na_2_CO_3_, and the absorbance value was measured using a UV–visible scanning spectrophotometer at 420 nm. The inhibition rate was expressed as a percentage using the following Formula (2):

Inhibition (%) = [1 − (negative control absorbance/experimental control absorbance)] × 100.(2)

### 2.5. Analysis of Fatty Acids

Fatty acids were analyzed as per Cho et al. [11]. A measure of 1 g of the sample powder was added into a test tube with 3 mL of 0.5 N methanolic NaOH, and the mixture was heated in a 100 °C heating block for 10 min. Then, 2 mL of boron trifluoride (BF_3_) was added, stirred, and heated again at 100 °C for 30 min. After the reaction, 1 mL of isooctane was mixed in; the isooctane layer was recovered and dehydrated with anhydrous sodium sulfate. Finally, the reaction samples were filtered through a 0.45 μm membrane filter and analyzed by GC. The mobile phase used nitrogen gas at a flow rate of 1 mL/min. The oven temperature was held at 140 °C for 5 min; then, it was increased by 20 °C/min up to 180 °C, where it was held for 2 min. It was further increased by 5 °C up to 230 °C and maintained for 35 min. The column utilized was an SP-2560 capillary column (100 m × 0.25 mm i.d., 0.25 μm film thickness, Sigma-Aldrich), with the injector temperature set at 220 °C, and the flame ion detector temperature at 240 °C.

### 2.6. Analysis of Free Amino Acids

For free amino acid analysis, 0.1 g of each sample was weighed in a test tube, and 5 mL of distilled water was added, stirred, and then hydrolyzed at 60 °C for 1 h. A volume of 1 mL of 10% 5-sulfosalicylic acid was added to the reaction mixture and incubated at 4 °C for 2 h to precipitate proteins. After centrifuging for 3 min, the supernatant was filtered through a 0.45 μm membrane filter. The filtrate was concentrated under a rotary evaporator at 60 °C and dissolved in 2 mL of lithium citrate buffer (pH 2.2), filtered with a 0.45 μm membrane filter, and analyzed. The free amino acid contents were determined using an automatic amino acid analyzer [11]. An ion exchange column (4.6 × 60 nm, Hitachi HPLC Pack Column, #2622PF Column, Hitachi High-Tech Science Corporation, Tokyo, Japan) was used. The detection used a UV detector which conducted measurements at 570 and 440 nm. The mobile phases of pump 1 and pump 2 were conducted by KANTO HITACHI High-Speed Amino Acid Analyzer PF-1, 2, 3, and 4 and RG and Wako Ninhydrin Coloring Solution Kit for HITACHI; flow rates of 0.35 and 0.3 mL/min were used, respectively, and the injection volume was 20 µL.

### 2.7. Analysis of TPCs, TFCs, and Isoflavone Derivatives

A measure of 40 mL of 50% ethanol was dispensed into 1 g of each sample and they were shaken for 12 h. After centrifugation at 6000 rpm for 15 min, the supernatant was filtered through a 0.45 μm membrane filter.

TPCs were calculated using the Folin–Denis method, as outlined by Lee et al. [5], and the absorbance of the solution was measured at 750 nm. TPCs were quantified using the equation procured from the gallic acid standard curve: TPCs = (Sample absorbance + 0.0038)/53.029. The method outlined by Lee et al. [5] was used to determine the TFCs. The absorbance value was measured at 420 nm, and the TFCs were determined using the formula derived from the rutin standard curve: TFCs = (Sample absorbance + 0.0013)/16.16.

The isoflavone derivative was analyzed using the method of Hwang et al. [12]. The column used for the analysis was a Lichrospher 100 RP C18 (5 μm, 4.6 × 250 mm, Merck, Darmstadt, Germany), and the column temperature was maintained at 30 °C. As mobile phase solvents, water with glacial acetic acid was used as solvent A and acetonitrile (ACN) was used as solvent B with glacial acetic acid. The sample injection amount was 20 μL and the mobile phase speed was maintained at 1 mL/min. Based on solvent B, it was analyzed at the following times and percentages: 0 min—0%; 25 min—20%; 35 min—25%; 45 min—35%; 50 min—35%. The assay absorbance was measured at 254 nm using a diode array detector. The isoflavone stock solution was diluted with DMSO to a concentration of 1 mg/mL, and calibration curves were sequentially prepared at seven concentrations (1, 5, 10, 25, 50, 75, and 100 μg/mL). In this process, we confirmed a high linearity with correlation coefficients (*r*^2^) > 0.998 for each curve.

### 2.8. Analysis of Anti-Obesity Effects

#### 2.8.1. Laboratory Animal Specifications

With the approval of the Namhae Garlic Research Institute Animal Research Ethics Committee (RNGRI-2018-1), 5-week-old C57BL/6J male mice with an average weight of 20 to 22 g were obtained from the central laboratory animal (Seoul, Republic of Korea), and they were fed on commercially available solid feed (Rat chow, Samyang Corp., Seoul, Republic of Korea) for 1 week. Then, in the 2nd week, male C57BL/6J mice (*n* = 24) were divided into four groups (*n* = 6 per each group) by the egg mass method so that the average weight of each experimental group was similar, and breeding was performed.

#### 2.8.2. Composition of the Experimental Group, Experimental Diet, and Feeding Amount of the Sample

The four groups (*n* = 6 per each group) were as follows: a normal diet control group (ND) that was provided with a standard diet, an HFD control group (HFD), a group fed HFD along with UFIESLs administration (HFD + UFIESL), and a group fed HFD with FIESLs administration group (HFD + FIESL). Normal and HFD fed to the experimental animals were prepared according to Table 1. Each experimental group received the corresponding sample in addition to the HFD according to Table 2. Given the small sample added, the basic diet composition ratio was not separately adjusted. The dose of 27 mg/day of isoflavones per 60 kg body weight is based on the daily intake recommendations set by Ministry of Food and Drug Safety [13].

#### 2.8.3. Dietary Intake, Dietary Efficiency, and Body Weight Measurements

During the experimental feeding period, the diet was fed every day at 5:00 p.m., and the remaining amount was examined at approximately 10:00 a.m. the next day. Fresh tap water was supplied fresh every day. Body weight was measured once a week at a regular time. To calculate dietary efficiency (%), the weight gain during the experiment was divided by the total food intake during the same period.

#### 2.8.4. Analysis of Epididymal Fat and Serum Biomarkers

After 10 weeks of experimental feeding and fasting for 16 h on the final day, inhalational anesthesia was administered for the small animals, and cardiac blood collection was conducted. The collected blood was cooled in ice water for 30 min and centrifuged at 3000 rpm for 15 min to separate the serum. The fat around the epididymis of the experimental animals was separated immediately after blood sampling, washed with physiological saline, dried with absorbent paper, and weighed.

The total serum total lipid content was calculated by dissolving 20 μL of serum with concentrated sulfuric acid and a phospho-vanillin reagent; this was then incubated at 37 °C for 15 min. Then, absorbance was measured at 540 nm using the sample-free group as a control, and it was substituted into a calibration curve prepared under the same conditions for each concentration using olive oil as a standard material [14]. Blood glucose and triglyceride levels, total cholesterol, and high-density lipoprotein cholesterol (HDL cholesterol) content in serum were measured using an automatic blood analyzer (DRICHAM 4000i, Tokyo, Japan). Leptin concentration in serum was measured by an enzyme-linked immunosorbent assay (ELISA) using a mouse ELISA kit (R&D Systems Inc., Minneapolis, MN, USA).

### 2.9. Analysis of Gut Microbiota

A sample of 1 g of the large intestine samples from each experimental group was homogenized with 10 mL of phosphate-buffered saline (pH 7.0) in a cool environment. After filtering the samples through sterile gauze, the liquid supernatant (3 mL) was centrifuged at 13,000 rpm for 5 min at 4 °C. To isolate metagenomic DNA, the complete pellets were submitted to DNeasy Powersoil Pro Kit (Qiagen, Hilden, Germany), and DNA extraction was performed according to the manufacturer’s protocol with slight modifications. Only the lysis step was modified, as follows: the pellets were suspended with 300 μL supernatant from the filtered samples; this was added to the Powerbead pro tube with 800 μL solution CD1, and vortexed for 10 min. Bacterial amplicon sequencing was performed according to the manufacturer’s instructions (Illumina, San Diego, CA, USA). Oligomers containing the bacterial specific sequence of 16S rRNA gene [5′-CCAGCAGCCGCGGTAATACG-3′ (518F, forward primer) and 5′-GACTACCAGGGTATCTAATCC-3′ (805R, reverse primer)] [15,16]; additionally, the Illumina overhang adapter sequence was used as an amplicon primer. Sample DNAs were PCR-amplified with an annealing temperature of 58 °C. The purified amplicons were PCR-indexed using an Illumina Nextera XT index kit. The purified library was quantified, pooled, and combined with the PhiX control (Illumina). The library was paired-end (151 bp × 2) sequenced using the iSeq 100 platform. Paired reads were first merged and then processed to remove short or low-quality sequences and potential chimeras. More than 50,000 quality-filtered reads were obtained from each sample. Operational taxonomic units (OTUs) were defined at the 97% sequence–identity cutoff using the VSEARCH algorithm [17]. Taxonomic assignment was conducted using the RDP classifier online (https://rdp.cme.msu.edu/classifier/) (accessed on 3 February 2023).

### 2.10. Statistical Analysis

The physicochemical properties, nutrient compounds, and enzyme inhibition activities are expressed as a mean ± standard deviation (SD). The Statistical Analysis System (SAS) software (ver. 9.4; SAS Institute, Cary, NC, USA) was used to perform an independent samples *t*-test or analysis of variance to determine significant differences among the groups. When differences among groups were confirmed, Duncan’s multiple range test was performed (*p* < 0.05). Non-metric multidimensional scaling (NMDS) plots and heatmaps were generated using R software (version 4.3.2). The correlation between gut microbiota and metabolite compounds was determined by Pearson’s correlation coefficient analyses in the correlation coefficient analyses in the corrplot package implemented in R version 4.3.2 [18].

## 3. Results and Discussion

### 3.1. Comparison of the Physicochemical Properties and Viable Cell Numbers in UFIESLs and FIESLs

Table 3 shows the physicochemical properties and viable cell numbers of UFIESLs and FIESLs. Before fermentation (UFIESLs), the pH, acidity, reducing sugar, and soluble protein were 4.0, 0.17%, 1.82 mg/g, and 7.01 mg/g, respectively. After fermentation (FIESLs), the pH decreased to 3.82, whereas the acidity, reducing sugar, and soluble protein increased to 0.19%, 2.75 mg/g, and 7.31 mg/g, respectively. In addition, in FIESLs the concentrations of *L. plantarum* P1201 and *L. brevis* BMK184 were 4.59 log CFU/g and 6.58 log CFU/g, respectively.

The decrease in pH and the increase in acidity after fermentation have been reported already, as has the fact that LAB cocultures could enhance the fermentation process [19]. In previous studies, plant material fermentation products from two LAB strains had decreased pH, acidity, and LAB cell number [5,12]. The decreased pH and increased acidity in FIESLs prevent the growth of spoilage microorganisms [20]. According to previous reports, several LABs consume reducing sugars and produce lactic acid during juice fermentation [21]. Conversely, FIESLs appear to contain higher reducing sugar levels before fermentation, in accordance with previous results [11]. The hydrolyzed amounts of reducing sugar from IESLs plant cells were higher than those converted into acid [22]. Meanwhile, IESLs were fermented with less inoculated *L. plantarum* P1201 than *L. brevis* BMK184, and the increase in acid in FIESLs was not great. This was due to the lower inoculation with *L. plantarum* P1201, which produces more lactic acid than *L. brevis* [23].

### 3.2. Fatty Acid Composition in UFIESLs and FIESLs

Table 4 shows the fatty acid contents of UFIESLs and FIESLs. There were no significant differences in the contents of saturated fatty acids (SFAs). The values were 349.3 (UFIESLs) and 341.1 (FIESLs) mg/100 g. Among SFAs, only lauric acid (C12:0) significantly increased from 4.90 to 8.20 mg/100 g after fermentation. The palmitic acid content was higher than that of other SFAs identified in UFIESLs and FIESLs. It decreased from 228.80 mg/100 g to 225.20 mg/100 g after fermentation, but the difference was not significant. In contrast, the content of unsaturated fatty acids (USFAs) significantly increased from 2170.80 mg/100 g before fermentation to 2363.10 mg/100 g after fermentation. USFAs such as oleic acid (C18:1) and linoleic acid (C18:2) significantly increased after fermentation. Especially, oleic acid and linoleic acid were the major USFAs components. Before fermentation, the content of oleic acid was 295.10 mg/100 g and that of linoleic acid was 1573.30 mg/100 g. After fermentation, the content increased to 337.90 mg/100 g for oleic acid and to 1720.00 mg/100 g for linoleic acid.

LAB are not notably recognized for their lipolytic activity. However, oleic acid and linoleic acid values increased after LAB fermentation, in line with previous results [24]. Further, lauric acid content was increased two-fold compared with UFIESLs. Certain LAB of apparently possess an internal system of lipase and esterase [25]. Accordingly, lauric acid concentration increased as well as that of oleic acid and linoleic acid in FIESLs with *L. brevis* and *L. plantarum*, we did not know how LAB strains perform with increasing lauric acid. Zhao et al. [26] reported that lauric acid has an anti-obesity effect and improved HFD-induced gut microbiota dysbiosis. It is suggested that a diet of FIESLs was able to increase anti-obesity effects and beneficial gut microbiota.

### 3.3. Free Amino Acid Composition in UFIESLs and FIESLs

Table 5 shows the free amino acid content of UFIESLs and FIESLs. Total amino acids, including non-essential amino acids (NEAAs) and essential amino acids (EAAs), significantly increased from 1316.43 to 4537.71 mg/100 g after fermentation. Among NEAAs, the content of aspartic acid increased most significantly from 99.04 to 394.85 mg/100 g after fermentation, making it the majority component. Conversely, the content of aspartic acid-NH_2_ decreased from 383.74 to 179.94 mg/100 g after fermentation. The content of all EAAs increased after fermentation. Particularly, valine content increased from 66.00 mg/100 g before fermentation to 80.06 mg/100 g after fermentation, a higher increase than other EAAs. Furthermore, isoleucine also increased from 41.73 mg/100 g to 54.27 mg/100 g after fermentation.

Amino acid components change with fermentation conditions. Consistent with a previous study, amino acids in IESLs differed between UFIESLs and FIESLs [5,12,27]. The arginine content, associated with bacterial growth considerably decreased after fermentation (84.18 → 0.73 mg/100 g). Many LABs use arginine and citrulline as energy sources through the arginine deiminase (ADI) pathway system. In fact, *L. brevis* appears to have arginine and citrulline catabolism pathways [28]. ADI has converted arginine into citrulline and ammonia, subsequently causing citrulline degradation and producing ornithine. According to this phenomenon, it is suggested that arginine converted into ornithine at that time arginine and citrulline declined and ammonia was also produced. Thus, citrulline was not detected in the FIESL, and ornithine levels significantly increased from 0.56 to 75.40 mg/100 g after fermentation.

### 3.4. Comparison of TPCs, TFCs, and Isoflavone Compositions in UFIESLs and FIESLs

**Figure 1:**
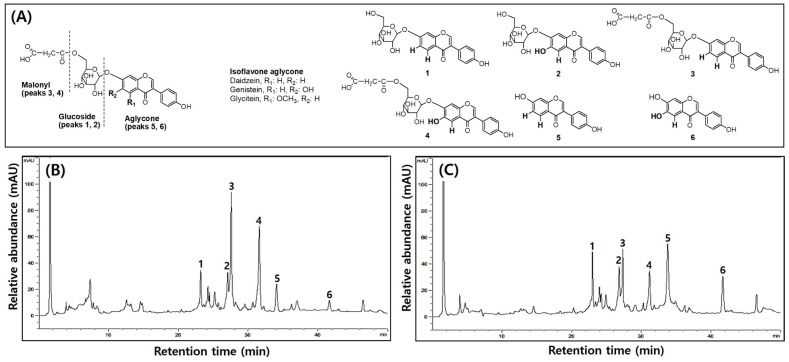
The chemical structure of six isoflavones and typical HPLC chromatogram of unfermented and fermented isoflavone-enriched soybean leaves. (**A**) Isoflavone chemical structures in soybean leaves; (**B**) unfermented isoflavone-enriched soy leaves; (**C**) fermented isoflavone-enriched soy leaves. 1—Daidzin; 2—Genistin; 3—Malnolyl-β-daidzin; 4—Malnolyl-β-genistin; 5—Daidzein; 6—Genistein.

Table 6 shows the TPCs, TFCs, and isoflavone contents of UFIESLs and FRESLs, and the isoflavone chemical structures and chromatogram are shown in Figure 1. The TPCs and TFCs increased significantly from 2.21 and 2.18 mg/g to 2.74 and 2.87 mg/g, whereas the total isoflavone content decreased from 2.64 to 2.35 mg/g. The contents of daidzin and genistin, which are glycosides, were 0.75 and 0.44 mg/g, respectively, before fermentation, decreasing after fermentation to 0.60 and 0.30 mg/g. In contrast, the contents of daidzein and genistein, which are non-glycosides, increased from 0.10 mg/g and 0.06 mg/g to 0.35 and 0.12 mg/g after fermentation.

Phenolic compounds are commonly present in plants and their levels typically increase through fermentation with microorganisms. In particular, LAB was generally used in the food industry as a fermentation microorganism because of its safety and diversity effect [29]. During fermentation of IESLs, TPCs, and TFCs levels slightly increased, as did those of isoflavone aglycones, while levels of glycosides and malonyl–glycosides decreased, in line with previous results [5]. This is explained because the glycosidases and decarboxylases produced by LAB release phenolic compounds from IESL plant cells [30]. Furthermore, the conversion of isoflavones from glycosides to aglycone enhanced their bioavailability [31].

### 3.5. Comparison of Digestive Enzyme Inhibitory Activities in UFIESLs and FIESLs

**Figure 2:**
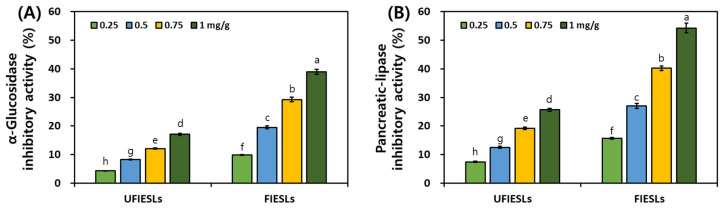
Digestive enzyme inhibitory activities of unfermented and fermented isoflavone-enriched soybean leaves. (**A**) α-Glucosidase inhibitory activity (%); (**B**) pancreatic–lipase inhibitory activity (%). Samples: UFIESLs—unfermented isoflavone-enriched soybean leaves; FIESLs—fermented isoflavone-enriched soybean leaves. Values of different letters are significantly different on the bars (*p* < 0.05).

Figure 2 shows the digestive enzyme inhibitory activities of UFIESLs and FIESLs. In UFIESLs, α-glucosidase and pancreatic lipase inhibitory activities increased with the sample concentration; this increase was further observed after fermentation. Specifically, at 1 mg/g, the inhibitory activities of α-glucosidase and pancreatic lipase significantly increased from 17.09% and 25.67% to 38.94% and 54.26%, respectively.

Phenolic compounds precipitate protein inhibiting digestive enzymes [32]. We confirmed changes in the inhibitory activities of α-glucosidase and pancreatic lipase after fermentation, as well as increased concentration of TPCs and TFCs, in line with previous results [5,33]. The inhibition of α-glucosidase and pancreatic lipase delays carbohydrate digestion and reduces lipid absorbance [34]. FIESLs may be useful for treating diabetes and may have anti-obesity effects.

### 3.6. Anti-Obesity Effects in UFIESLs and FIESLs

**Figure 3:**
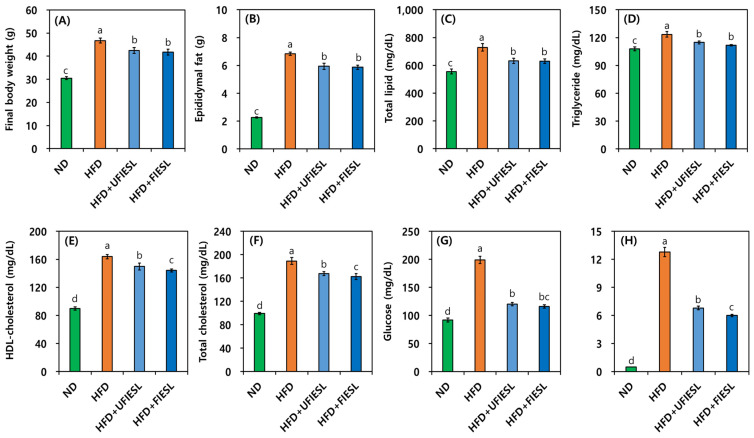
Comparison of body weight, epididymal fat, and serum biomarkers by the diet of unfermented and fermented isoflavone-enriched soybean leaves. (**A**) Final body weight; (**B**) epididymal fat; (**C**) total lipid; (**D**) triglyceride; (**E**) HDL cholesterol; (**F**) total cholesterol; (**G**) glucose; (**H**) leptin. Samples: ND, normal diet; HFD, high-fat diet; HFD + UFIESL, HFD and unfermented isoflavone-enriched soybean leaves; HFD + FIESL, HFD and fermented isoflavone-enriched soybean leaves. All values are presented as the mean ± SD (*n* = 6). Values of different letters are significantly different on the bars (*p* < 0.05).

Figure 3 shows the anti-obesity effects feeding an HFD model with UFIESLs and FIESLs. Figure 3A,B show the final weight and epididymal fat according to dietary condition. The final weights of the ND and HFD groups were 30.50 and 46.75 g. The final weight of the HFD + UFIESL group was 42.50 g, which was slightly higher than that of the HFD + FIESL group (41.75 g), which received FIESLs. The epididymal fat weights in the ND and HFD groups were 2.26 and 6.84 g, respectively. The weights of epididymal fat in the HFD + UFIESL group and HFD + FIESL group were 5.94 and 5.87 g, respectively, showing a similar trend to that of the final weight. Figure 3C–F show the total lipid, triglyceride, HDL cholesterol, and total cholesterol results in each group. The HFD group showed significantly higher total lipid, triglyceride, HDL cholesterol, and total cholesterol values than the ND group (555.44, 107.83, 89.83, and 99.17 mg/dL → 729.00, 123.50, 163.83, and 189.17 mg/dL). Dietary intake of UFIESLs and FIESLs decreased in the total lipid, triglyceride, HDL cholesterol, and total cholesterol levels. The HFD + FIESL (629.56, 111.83, 144.17, and 162.50 mg/dL) group displayed lower total lipid, triglyceride, HDL cholesterol, and total cholesterol contents than the HFD + UFIESL group. The blood glucose and leptin levels are shown in Figure 3G,H. Glucose levels were lower in the HFD + UFIESL and HFD + FIESL than in the HFD group (199.17 → 120.17 and 116.00 mg/dL). In addition, the HFD + UFIESL and the HFD + FIESL group presented decreased leptin values compared with the HFD (12.79 μg/dL) group, and the HFD + FIESL (5.99 μg/dL) group exhibited lower leptin levels than HFD + UFIESL (6.79 μg/dL) group.

Both mature and immature SLs have anti-obesity effects due to kaempferol and coumestrol [2,35]. Additionally, Choi et al. [36] reported that SLs reduced body fat content and waist circumference, while lowering plasma triglyceride levels, in overweight patients. Additionally, Xie et al. [37] reported that IESLs prevent menopause associated obesity. However, the anti-obesity effects of fermented SLs and fermented IESLs have not been reported till now; this is the first study to compare and evaluate the anti-obesity effects of UFIESLs and FIESLs. Fermented foods, such as *mulberry* leaves [38], *Gochujang* [39], barley [40], celery [41], and fruits [42], with diverse microorganism strains [43], typically have stronger anti-obesity effects due to their production of organic acids, free amino acids, phenolic compounds, and isoflavones (daidzein and genistein) during fermentation [38,39,40,41,42,43]. Particularly, phenolic compounds in fermented plants are considerably correlated with anti-obesity effects [43]. Alternatively, Zhao et al. [26] reported that lauric acid has a regulating effect on hyperlipidemia. In this study, FIESLs were enriched compounds such as lauric acid, TPCs, TFCs, daidzein, and genistein, and the HFD + FIESL group showed improvements in body weight, epididymal fat, and serum biomarkers due to the increase in these substances associated with anti-obesity, in line with previous reports [38,39,40,41,42,43].

### 3.7. Gut Microbiota Distribution in UFIESLs and FIESLs

**Figure 4:**
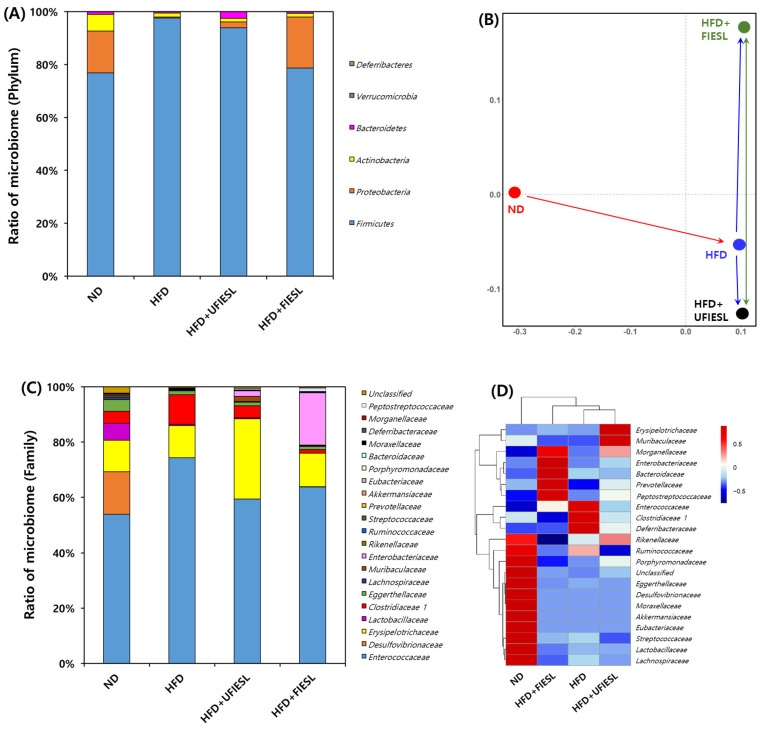
Correlation with the distribution of gut microbiota by the diet of unfermented and fermented isoflavone-enriched soybean leaves. (**A**) Ratio of microbiome (Phylum); (**B**) non-metric multidimensional scaling (NMDS) plot using gut microbiota for bacteria; (**C**) ratio of microbiome (family); (**D**) heatmap comparison and hierarchical clustering dendrogram based on the relative abundance of gut microbiota (family). Samples: ND, normal diet; HFD, high-fat diet; HFD + UFIESL, HFD and unfermented isoflavone-enriched soybean leaves; HFD + FIESL, HFD and fermented isoflavone-enriched soybean leaves.

We analyzed the microbiota composition of all experimental groups at the phylum level (Figure 4A). The microbial community of the ND group mainly included firmicutes (76.88%), proteobacteria (15.79%), and actinobacteria (6.30%). In the HFD group, firmicutes dominated with a staggering 97.63%. UFIESLs and FIESLs led to changes in the composition of the microbiota, such as a reduction in firmicutes and an increase in proteobacteria compared with the microbiota composition of the HFD group. In particular, the main community composition of the HFD + FIESL group was similar to that of the ND group. In the HFD + FIESL group, firmicutes accounted for 78.65%, proteobacteria 19.32%, and actinobacteria 1.31%. Results from the NMDS at the phylum level are shown in Figure 4B. The difference in the community composition between the ND and HFD groups was indicated with a red arrow. When UFIESLs or FIESLs were fed to the HFD model, differences in community composition (indicated by the green arrow) due to fermentation, was shown to be greater than the distance (indicated by the blue arrow) between the HFD and either the HFD + UFIESL or the HFD + FIESL communities. This suggests that IESL fermentation can induce changes in the gut microbiota composition.

Figure 4C,D show the relative abundance and heatmap of each group at the family levels. The HFD group displayed higher values of *Enterococcaceae* and *Clostridiaceae* 1 than the ND group (53.85 and 4.42% → 74.31 and 10.64%). However, the three HFD groups had a lower ratio of *Desulfovibrionaceae* than the ND group (15.43%). The *Enterococcaceae* and *Clostridiaceae* 1 proportions in the HFD + UFIESL group (59.34% and 4.37%) and the HFD + FIESL group (63.76% and 12.18%) were lower than those in the HFD group. Interestingly, *Enterobacteriaceae* was only abundant in the HFD + FIESL group. According to the heatmap, there were distinct differences between groups at the family level. Nevertheless, the HFD + FIESL group was the closest to the ND group, and the HFD + UFIESL group was closest to the HFD group.

**Figure 5:**
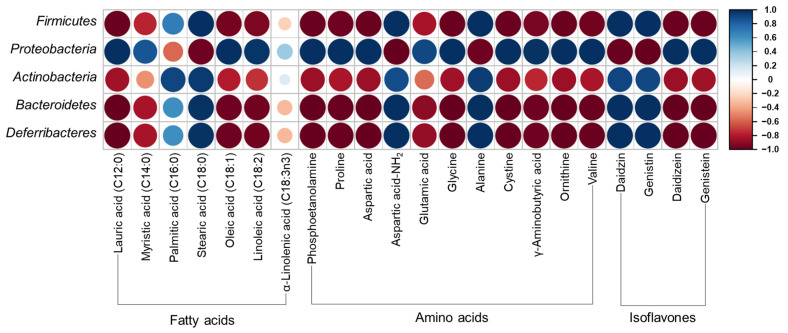
Pearson’s correlation matrix calculated from relative abundance of gut microbiota and selected high-content metabolite compounds. The red and blue colors correspond to negative correlations and the positive correlations, respectively. The size of each circle and color intensity are proportional to the correlation coefficient. (For interpretation of the references to color in this figure legend, the reader is referred to the web version of this article).

Figure 5 shows Pearson’s correlation matrix calculated from the relative abundance of gut microbiota and selected high-content metabolite compounds. *Proteobacteria* showed a high positive correlation with lauric acid, myristic acid, oleic acid, linoleic acid, phosphoetanolamine, proline, aspartic acid, glutamic acid, glycine, cystine, γ-aminobutyric acid, ornithine, valine, daidizein, and genistein. *Firmicutes*, *Actinobacteria*, *Bacteroidetes,* and *Deferribacteres* showed a contrary correlation with *Proteobactera*, that is, a negative correlation with the aforementioned compounds.

*Firmicutes* and *Bacteroidetes* are the most abundant phyla in the human gut microbiota contributing to the development of obesity, whereas some *Bacteroidetes* are considered anti-obesity microbiota [9,44]. *Firmicutes* levels increased with HFD, in accordance with previous studies [41,42]. *Bacteroidetes* levels were higher in HFD + UFIESL than in the ND group. Recent findings have shown that *Firmicutes* and Bacteroidetes are positively related to obesity development [44]. Meanwhile, Johnson et al. [45] reported that *Bacteroidetes* produce SCFAs in the gut, and Lau et al. [46] and Shang et al. [47] found lower *Bacteroidetes* levels in the guts of obese mice but were abundant in the guts of lean women, suggesting a negative association with obesity. Dietary intake of FIESLs decreased *Firmicutes* values in the HFD + FIESL group. We confirmed a positive correlation between firmicutes and stearic acid, aspartic acid-NH_2_, daidzin, and genistein (*r* = 1.00, *p* = 0.000). Conversely, the levels of proteobacteria, which decreased with the HFD diet, were restored to ND group levels by the FIESLs diet. *Proteobacteria* levels showed a significant positive correlation with lauric acid, phosphoetanolamine, proline, aspartic acid, glycine, cystine, ornithine, valine, daidzein, and genistein (*r* = 1.00, *p* = 0.000). Next, we investigated whether *Proteobacteria* were associated with anti-obesity, in contrast with previous results [48]. Although previous studies reported *Bacteroidetes* as obesity-inducing microbiota, like some specific *Bacteroidetes* which have been reported as anti-obesity microbiota, *Proteobacteria* may be considered as anti-obesity microbiota in this study. Therefore, FIESLs could repair dysbiosis gut microbiota structure in the HFD groups. However, specific species of *Proteobacteria* are probably involved in anti-obesity effect; to identify the specific species of *Proteobacteria* involved in this effect, further research is needed.

## 4. Conclusions

FIESLs demonstrated an anti-obesity effect against an HFD by regulating body weight, epididymal fat, blood glucose, leptin, and serum biomarkers such as total lipid, triglyceride, and total cholesterol, in addition to modulating gut microbiota composition. Fermentation of IESLs with LAB enhanced the levels of fatty acids, free amino acids, TPCs, TFCs, and isoflavone aglycones. The enrichment in such metabolite may contribute to its the efficacy on digestive enzyme inhibition and against obesity. Furthermore, FIESLs improved gut microbiota dysbiosis. Some gut microbiota have a strong positive correlation with several anti-obesity substances, suggesting that FIESLs play a regulatory role in gut microbiota associated with diseases induced by an HFD. Therefore, FIESLs could be considered functional foods, with beneficial effects for the prevention and treatment of obesity. However, further research is required to investigate the improvement of the gut microbiota community and anti-obesity effects through the administration of substances that show a positive correlation either individually or in combination. In addition, further research is deemed necessary to support the claim that *Proteobacteria* are anti-obesity-related microbes.

## Figures and Tables

**Table 1:** Diet compositions in experimental groups.

Ingredients	Groups (g/100 g Diet)	
	ND	HFD
Corn starch	15	15
Casein	20	20
Cellulose	5	5
Sucrose	50	15
Vitamin mix ^a^	1	1
Mineral mix ^b^	3.5	3.5
L-Cysteine	0.3	0.3
Choline bitartrate	0.2	0.2
Soybean oil	5	5
Lard	-	35

^a^ AIN-93 Vitamin mixture. ^b^ AIN-93 Mineral mixture.

**Table 2:** Compositions of experimental groups and daily dosage of experimental diet.

Groups ^a^	Sample	Daily Dosage
HFD + UFIESL	Powder of unfermented IESLs	Sample concentration included isoflavone 27 mg/day/60 kg body weight
HFD + FIESL	Powder of fermented IESLs	Sample concentration included isoflavone 27 mg/day/60 kg body weight

^a^ Groups: HFD + UFIESL—HFD and unfermented isoflavone-enriched soybean leaves; HFD + FIESL—HFD and fermented isoflavone-enriched soybean leaves.

**Table 3:** Comparison of physicochemical properties, viable cell numbers, and digestive enzyme inhibitory activities of unfermented and fermented isoflavone-enriched soy leaves.

Item ^a^	Samples ^b^	
	UFIESLs	FIESLs
Physicochemical properties		
pH	4.00 ± 0.20 ^a^	3.82 ± 0.14 ^a^
Acidity (%, as lactic acid)	0.17 ± 0.00 ^a^	0.19 ± 0.01 ^a^
Reducing sugar (mg/g)	1.82 ± 0.04 ^b^	2.75 ± 0.12 ^a^
Soluble protein (mg/g)	7.01 ± 0.28 ^a^	7.31 ± 0.29 ^a^
Viable cell numbers (log CFU/g)		
*Lactiplantibacillus plantarum* P1201	nm ^c^	4.59 ± 0.15 ^a^
*Levilactobacillus brevis* BMK184	nm	6.58 ± 0.18 ^a^
Total	nm	11.17 ± 0.23 ^a^

^a^ All values are presented as the mean ± SD (*n* = 5). Values of different letters are significantly different in the same row (*p* < 0.05). ^b^ Samples: UFIESLs—unfermented isoflavone-enriched soybean leaves; FIESLs—fermented isoflavone-enriched soybean leaves. ^c^ nm: not measured.

**Table 4:** Comparison of fatty acid contents of unfermented and fermented isoflavone-enriched soybean leaves.

Contents (mg/100 g) ^a^	Samples ^b^	
	UFIESLs	FIESLs
Saturated fatty acids (SFAs)		
Lauric acid (C12:0)	4.90 ± 0.15 ^b^	8.20 ± 0.29 ^a^
Myristic acid (C14:0)	8.80 ± 0.14 ^a^	9.10 ± 0.31 ^a^
Palmitic acid (C16:0)	228.80 ± 5.83 ^a^	225.20 ± 13.61 ^a^
Stearic acid (C18:0)	84.30 ± 3.44 ^a^	76.00 ± 1.11 ^b^
Arachidic acid (C20:0)	6.60 ± 0.18 ^a^	6.60 ± 0.27 ^a^
Behenic acid (C22:0)	8.80 ± 0.46 ^a^	8.90 ± 0.36 ^a^
Lignoceric acid (C24:0)	7.10 ± 0.24 ^a^	7.10 ± 0.22 ^a^
Total	349.3 ± 5.88 ^a^	341.1 ± 18.06 ^a^
Unsaturated fatty acids (USFAs)		
Palmitoleic acid (C16:1)	2.40 ± 0.10 ^a^	2.80 ± 0.14 ^a^
Oleic acid (C18:1)	295.10 ± 9.58 ^b^	337.90 ± 7.10 ^a^
Linoleic acid (C18:2)	1573.70 ± 33.35 ^b^	1720.00 ± 117.84 ^a^
γ-Linolenic acid (C18:3n6)	2.20 ± 0.05 ^a^	2.20 ± 0.08 ^a^
α-Linolenic acid (C18:3n3)	294.80 ± 6.06 ^a^	297.20 ± 6.66 ^a^
Eicosadienoic acid (C20:2)	2.60 ± 0.09 ^ab^	3.00 ± 0.09 ^a^
Total	2170.80 ± 28.17 ^a^	2363.10 ± 114.94 ^a^
Total fatty acids (TFAs)	2520.10 ± 26.23 ^a^	2704.20 ± 101.14 ^a^

^a^ All values are presented as the mean ± SD (*n* = 5). Values of different letters are significantly different in the same row (*p* < 0.05). ^b^ Samples: UFIESLs—unfermented isoflavone-enriched soybean leaves; FIESLs—fermented isoflavone-enriched soybean leaves.

**Table 5:** Comparison of free amino acid contents of unfermented and fermented isoflavone-enriched soybean leaves.

Contents (mg/100 g) ^a^	Samples ^b^	
	UFIESLs	FIESLs
Non-essential amino acids (NEAAs)		
Phosphoetanolamine	nd ^c^	11.37 ± 0.59 ^a^
Proline	113.92 ± 7.19 ^b^	144.41 ± 9.13 ^a^
Hydroxyproline	4.96 ± 0.26 ^a^	nd
Aspartic acid	99.04 ± 5.68 ^b^	394.85 ± 13.18 ^a^
Serine	38.64 ± 2.38 ^b^	50.56 ± 1.22 ^a^
Aspartic acid-NH_2_	383.74 ± 20.33 ^a^	179.94 ± 8.08 ^b^
Glutamic acid	73.60 ± 1.82 ^a^	76.85 ± 2.44 ^a^
Aminoadipic acid	11.56 ± 0.51 ^b^	20.46 ± 1.11 ^a^
Glycine	9.03 ± 0.56 ^b^	19.89 ± 1.12 ^a^
Alanine	65.86 ± 2.64 ^a^	57.97 ± 3.60 ^a^
Citrulline	1.32 ± 0.04 ^a^	nd
α-aminobutyric acid	14.98 ± 0.56 ^a^	15.37 ± 0.84 ^a^
Cystine	nd	6.95 ± 0.33 ^a^
Tyrosine	23.60 ± 1.68 ^a^	1.99 ± 0.13 ^b^
β-alanine	12.62 ± 0.48 ^a^	18.48 ± 1.46 ^a^
β-aminoisobutyric acid	7.43 ± 0.25 ^a^	7.10 ± 0.25 ^a^
γ-aminobutyric acid	112.84 ± 5.20 ^a^	126.01 ± 5.75 ^a^
Aminoethanol	5.04 ± 0.23 ^a^	6.60 ± 0.15 ^a^
Hydroxylysine	1.25 ± 0.08 ^a^	nd
Ornithine	0.56 ± 0.03 ^b^	75.40 ± 2.29 ^a^
Arginine	84.18 ± 3.94 ^a^	0.73 ± 0.05 ^b^
Total	1064.17 ± 15.43 ^b^	1214.93 ± 23.72 ^a^
Essential amino acids (EAAs)		
Threonine	26.35 ± 1.00 ^b^	40.95 ± 1.55 ^a^
Valine	66.00 ± 2.38 ^b^	80.06 ± 4.23 ^a^
Methionine	3.43 ± 0.14 ^a^	4.09 ± 0.18 ^a^
Isoleucine	41.73 ± 1.85 ^b^	54.27 ± 1.87 ^a^
Leucine	36.39 ± 1.16 ^b^	45.75 ± 1.78 ^a^
Phenylalanine	39.44 ± 2.50 ^b^	53.12 ± 1.88 ^a^
Lysine	20.28 ± 0.75 ^a^	21.75 ± 1.67 ^a^
Histidine	18.64 ± 0.93 ^a^	22.80 ± 0.81 ^a^
Total	252.26 ± 4.57 ^b^	322.79 ± 3.28 ^a^
Total amino acids (TAAs)	1316.43 ± 11.75 ^b^	1537.72 ± 23.56 ^a^
Ammonia	13.01 ± 0.81 ^b^	46.04 ± 1.41 ^a^

^a^ All values are presented as the mean ± SD (*n* = 5). Values of different letters are significantly different in the same row (*p* < 0.05). ^b^ Samples: UFIESLs—unfermented isoflavone-enriched soybean leaves; FIESLs—fermented isoflavone-enriched soybean leaves. ^c^ nd: not detected.

**Table 6:** Comparison of total phenolic, total flavonoid, and isoflavone contents of unfermented and fermented isoflavone-enriched soybean leaves.

Contents (mg/g) ^a^	Samples ^b^	
	UFIESLs	FIESLs
Total phenolic contents (TPCs)	2.21 ± 0.08 ^b^	2.74 ± 0.16 ^a^
Total flavonoid contents (TFCs)	2.18 ± 0.08 ^b^	2.87 ± 0.06 ^a^
Total isoflavone contents (TICs)	2.64 ± 0.04 ^a^	2.35 ± 0.02 ^b^
Daidzin	0.75 ± 0.05 ^a^	0.60 ± 0.01 ^b^
Genistin	0.44 ± 0.01 ^a^	0.30 ± 0.01 ^b^
Malnolyl-β-daidzin	0.73 ± 0.04 ^a^	0.56 ± 0.02 ^b^
Malnolyl-β-genistin	0.56 ± 0.02 ^a^	0.42 ± 0.01 ^b^
Daidizein	0.10 ± 0.01 ^b^	0.35 ± 0.01 ^a^
Genistein	0.06 ± 0.00 ^b^	0.12 ± 0.00 ^a^

^a^ All values are presented as the mean ± SD (*n* = 5). Values of different letters are significantly different in the same row (*p* < 0.05). ^b^ Samples: UFIESLs—unfermented isoflavone-enriched soybean leaves; FIESLs—fermented isoflavone-enriched soybean leaves.

## Data Availability

The original contributions presented in the study are included in the article, further inquiries can be directed to the corresponding authors.
